# Effects of Paeonol on Anti-Neuroinflammatory Responses in Microglial Cells

**DOI:** 10.3390/ijms16048844

**Published:** 2015-04-21

**Authors:** Chingju Lin, Hsiao-Yun Lin, Jia-Hong Chen, Wen-Pei Tseng, Pei-Ying Ko, Yu-Shu Liu, Wei-Lan Yeh, Dah-Yuu Lu

**Affiliations:** 1 Department of Physiology, School of Medicine, China Medical University, Taichung 40402, Taiwan; E-Mail: clin33@mail.cmu.edu.tw; 2 Graduate Institute of Neural and Cognitive Sciences, China Medical University, Taichung 40402, Taiwan; E-Mail: lingirl831@hotmail.com; 3 Department of General Surgery, Taichung Tzu Chi Hospital, Buddhist Tzu Chi Medical Foundation, Taichung 42743, Taiwan; E-Mail: guns5150@ms27.hinet.net; 4 Graduate Institute of Sports and Health, National Changhua University of Education, Changhua 500, Taiwan; E-Mail: twp@cc.ncue.edu.tw; 5 Department of Medical Laboratory Science and Biotechnology, China Medical University, Taichung 40402, Taiwan; E-Mail: codo710011@gmail.com; 6 Graduate Institute of Basic Medical Science, China Medical University, Taichung 40402, Taiwan; E-Mail: yushuliu220@gmail.com; 7 Department of Cell and Tissue Engineering, Changhua Christian Hospital, Changhua 500, Taiwan; E-Mail: ibizayeh0816@hotmail.com; 8 Department of Photonics and Communication Engineering, Asia University, Taichung 40402, Taiwan

**Keywords:** paeonol, microglia, neuroinflammation, AMPK, GSK 3α/β

## Abstract

Increasing studies suggest that inflammatory processes in the central nervous system mediated by microglial activation plays an important role in numerous neurodegenerative diseases. Development of planning for microglial suppression is considered a key strategy in the search for neuroprotection. Paeonol is a major phenolic component of Moutan Cortex, widely used as a nutrient supplement in Chinese medicine. In this study, we investigated the effects of paeonol on microglial cells stimulated by inflammagens. Paeonol significantly inhibited the release of nitric oxide (NO) and the expressions of inducible nitric oxide synthase (iNOS) and cyclooxygenase-2 (COX-2). Treatment with paeonol also reduced reactive oxygen species (ROS) production and inhibited an ATP-induced increased cell migratory activity. Furthermore, the inhibitory effects of neuroinflammation by paeonol were found to be regulated by phosphorylated adenosine monophosphate-activated protein kinase-α (AMPK-α) and glycogen synthase kinase 3 α/β (GSK 3α/β). Treatment with AMPK or GSK3 inhibitors reverse the inhibitory effect of neuroinflammation by paeonol in microglial cells. Furthermore, paeonol treatment also showed significant improvement in the rotarod performance and microglial activation in the mouse model as well. The present study is the first to report a novel inhibitory role of paeonol on neuroinflammation, and presents a new candidate agent for the development of therapies for inflammation-related neurodegenerative diseases.

## 1. Introduction

Microglial cells, the major resident immune cells in the central nervous system (CNS), play a pivotal role in the first line of host defense by removing dead cells or pathogens [1]. The quiescent microglia cells are ramified. Upon microbial infections or CNS injuries, activated microglia cells can change into an amoeboid morphology and release pro- and anti-inflammatory mediators [2,3,4]. Although the activation of microglia is necessary for maintaining normal neuronal cell functions and tissue repair, the control of anti- and pro-inflammation mediators released is like a double-edged sword that needs to be tightly regulated [5]. Emerging studies have suggested that over-production of the proinflammatory mediators by activated microglia cells are associated with the pathogenesis of a variety of neurodegenerative diseases, including Alzheimer’s disease (AD) [6], Huntington’s disease [7], Parkinson’s disease (PD) [8,9], stroke [5] and hypobaric hypoxia [10]. For example, studies on the brains of the postmortem PD patients have shown the presence of activated microglia cells in the substantia nigra pars compacta [11] and revealed an elevation of inducible nitric oxide synthase (iNOS) and cyclooxygenase-2 (COX-2) expression in the striatum area [12]. Moreover, activated microglia cells also caused expression of iNOS and COX-2. It has been reported that synthesis of iNOS which continually generates high levels of nitric oxide (NO) [13], and induction of COX-2 expression [14] were closely correlated with the progression of neurodegeneration. In agreement with this notion, compounds with anti-neuroinflammation effects have been proposed to be potential therapeutic drugs in treating neurological diseases [15,16,17].

Adenosine monophosphate-activated protein kinase (AMPK) could be activated by upstream kinases like live kinase B1 (LKB1) or Calcium/calmodulin-dependent kinase kinase (CaMKK) through phosphorylation at the Thr^172^ on the α subunit [18,19]. AMPK has been reported to be involved in various biological functions, but the overall consequences depend on the degree of activation, cell types and the specific metabolic status of the cell. AMPK activation increased survival in cultured rat hippocampal neurons subjected to metabolic stress [20], while it led to neuronal apoptosis in human undifferentiated neuroblastoma cells [21]. Although the functions of AMPK in metabolism have been well studied, reports on the role of AMPK in neuro-inflammation still remain to be elucidated.

Paeonol, a major phenolic component of Moutan Cortex, the root bark of Paeonia moutan, is widely used as a nutrient supplement in Chinese medicine. It possess a broad range of properties like inhibiting collagen-induced platelet aggregation [22] and attenuating inflammatory responses in airways, coronary arteries, macrophages and microglia cells [23,24,25]. Accumulating evidence indicates that paeonol may be a promising neuroprotective or anti-neurodegenerative compound because of its anti-inflammatory and free-radical scavenging properties; paeonol protected neurons from oxygen-glucose deprivation-induced injuries [26] and from neurotoxicity caused by H_2_O_2_ treatment [27]. Moreover, paeonol reduced cerebral infarction involving the superoxide anion and microglia activation in ischemia-reperfusion injured rats [28]. The functions of paeonol might be associated with regulating production of proinflammation molecules and oxidative stresses. Chou [29] reported that the mechanisms by which paeonol exerted its anti-inflammatory and analgesic effects may be involved with decreased production of proinflammatory cytokines, NO and PGE_2_ and increased production of IL-10, an anti-inflammatory cytokine. In addition, in the model of carrageenan-injected rat paws, attenuation of the elevated iNOS and COX-2 protein expression, as well as neutrophil infiltration may also be mediated by the beneficial effects of paeonol administration [29]. A recent study by Tseng *et al.* [30] indicated that paeonol attenuated LPS-induced inflammation responses in primary microglia cells and protected cortical neuron cells from oxidative stress caused by 6-hydroxydopamine (6-OHDA) treatment. These effects were associated with attenuating overexpression of iNOS and COX-2, reducing ROS production and increasing superoxide dismutase activities [30]. Another study implied that inhibition of NF-κB translocation to the nucleus and suppression of the mitogen activated protein (MAP) kinase activities were involved in the anti-neuroinflammatory effects of paeonol [23]. Nevertheless, with its broad range of functions, mechanisms underlying paeonol’s effects may be intricate and need to be elucidated. Our study examined whether paeonol could reduce inflammatory molecules in microglial cells, and whether paeonol could alter the sickness behavior response to LPS. We found that paeonol effectively reduces neuroinflammatory and anti-oxidant effects through activating AMPKα and GSK 3α/β, and the protective effect of paeonol rescued inflammatory-mediated motor dysfunction and microglial activation in animal model.

## 2. Results

### 2.1. Paeonol Suppresses LPS/IFN-γ-Induced Inflammatory Responses in Microglia

**Figure 1:**
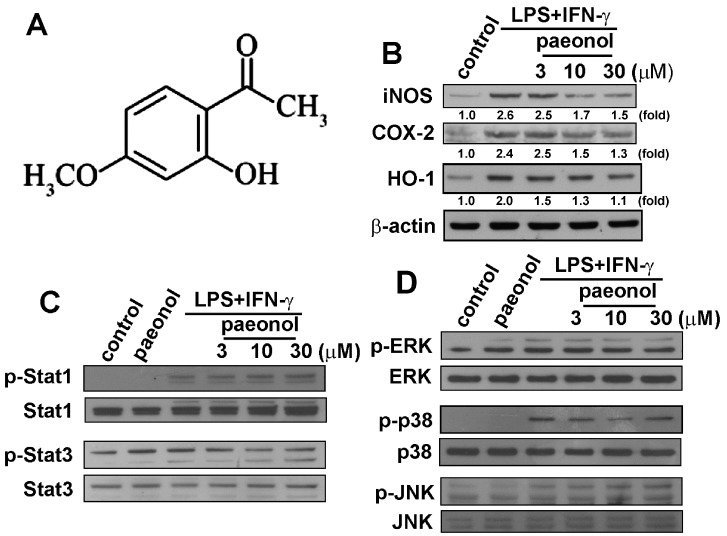
Effects of paeonol on inflammatory responses in BV-2 microglia. (**A**) The chemical structure of paeonol; (**B**) Cells were pretreated with various concentrations of paeonol (3, 10, or 30 μM) for 30 min before stimulation with LPS (10 ng/mL)/IFN-γ (10 ng/mL) for another 24 h. Whole-cell lysates were subjected to western blot analysis for iNOS, COX-2 and HO-1; (**C**,**D**) Cells were pretreated with various concentrations of paeonol (3, 10, or 30 μM) for 30 min before stimulation with LPS (10 ng/mL)/IFN-γ (10 ng/mL) for 90 min. Whole-cell lysates were subjected to western blot analysis using antibodies against the phosphorylated Stat1 and Stat3 (**B**), ERK1/2, p38 and JNK (**C**). Similar results were obtained for at least three independent experiments.

We used microglial cells to study the anti-neuroinflammatory mechanism of paeonol (Figure 1A). To determine the effect of paeonol on iNOS, COX-2 and HO-1 protein levels, cells were treated with LPS plus IFN-γ plus paeonol, and protein levels were detected using western blotting (Figure 1B). We further investigated the inhibitory effects of paeonol on STAT and MAP kinase signaling. As shown in Figure 1C, paeonol antagonized LPS/IFN-γ-induced STAT3 phosphorylation but not STAT1 phosphorylation. Moreover, paeonol also mildly reduced LPS/IFN-γ-induced p38 activation, but not ERK and JNK phosphorylation (Figure 1D). In addition, according to a cell viability assay, the various concentrations of paeonol used did not affect microglial cell death.

### 2.2. Paeonol Inhibits Migratory Activity and ROS Production in Microglial Cells

**Figure 2:**
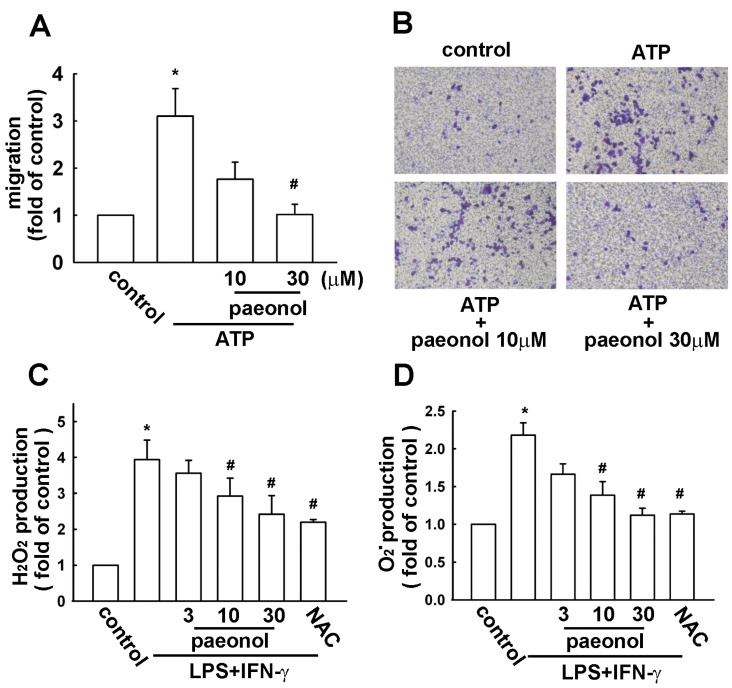
Effects of paeonol on cell migratory activity and ROS production in BV-2 microglia. (**A**) Cells were pretreated with paeonol (10 or 30 μM) for 30 min followed by stimulation with ATP (300 μM) for 24 h. *In vitro* migratory activities were examined using a cell transwell insert system. The results are expressed as means ± SEM of three independent experiments; The migrated cells were visualized by phase-contrast imaging (**B**); (**C**,**D**) Cells were pretreated with paeonol (3, 10, or 30 μM) or *N*-acetylcysteine (NAC) (1 mM) for 30 min followed by stimulation with LPS (10 ng/mL)/IFN-γ (10 ng/mL) for 2 h, the production of reactive oxygen species (ROS) were examined by flow cytometry; H_2_O_2_ and O_2_^−^ generation were determined using the fluorescence probes DCFH-DA (**C**) and DHE (**D**), respectively. Results are expressed as the mean ± SEM of four independent experiments. * *p* < 0.05 compared with the control group. ^#^ *p* < 0.05 compared with the LPS/IFN-γ-treated group.

As shown in Figure 2A, ATP significantly increased cell migration in microglial cells. However, the ATP-enhanced migratory activity was effectively reduced by paeonol (Figure 2A). The photographs of migrating cells are shown in Figure 2B. Next, we then used flow cytometry to evaluate the intracellular H_2_O_2_ and O_2_^−^ formation by using a fluorescent sensitive probe DCFH-DA and DHE. LPS plus IFN-γ induced a significant increase of DCFH-DA and DHE fluorescence, reflecting the increase of ROS. LPS plus IFN-γ treatment alone for 2 h induced approximately 4.0- and 2.2-fold increases in H_2_O_2_ and O_2_^−^ levels, respectively. However, treatment with paeonol concentration-dependently decreased H_2_O_2_ (Figure 2C) and O_2_^−^ (Figure 2D) production. In addition, H_2_O_2_ and O_2_^−^ levels were reduced by a ROS scavenger *N*-acetylcysteine as well (Figure 2C,D).

### 2.3. Anti-Neuroinflammatory Effects of Paeonol through AMPK Signaling Pathway

**Figure 3:**
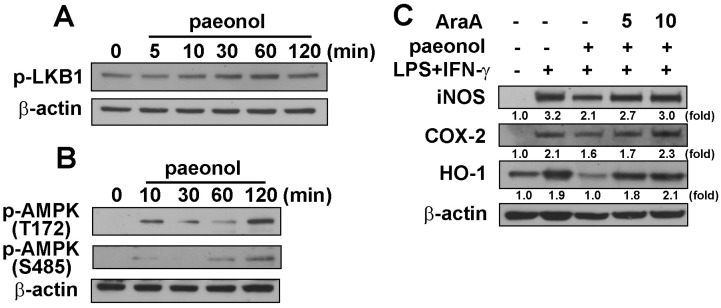
Involvement of AMPK by paeonol in neuroinflammation. BV-2 microglia cells were incubated with paeonol (10 μM) for indicated time periods (0–120 min). Whole-cell lysates were subjected to western blot analysis using antibodies against the phosphorylated LKB1 (Ser^428^) (**A**); and phosphorylated AMPKα at Thr^172^ and Ser^485^ (**B**); (**C**) Cells were pretreated with AraA (5 or 10 μM) for 30 min and incubated with paeonol (10 μM) for another 30 min before LPS (10 ng/mL)/IFN-γ (10 ng/mL) treatment for 24 h; Whole cell lysis proteins were extracted and subjected to western blot for iNOS, COX-2 and HO-1 (**C**). Similar results were obtained from at least four independent experiments.

We further determined the signaling pathway involved in anti-neuroinflammatory effects of paeonol. Stimulation of cells with paeonol increased the phosphorylation of AMPK upstream molecular regulators, LKB1 (Ser^428^), within a transient period (Figure 3A). Paeonol also increased AMPK phosphorylation at either the Thr^172^ or Ser^485^ sites (Figure 3B). In the presence of AMPK inhibitor, AraA [31], the inhibitory effects of paeonol on LPS plus IFN-γ induced iNOS, COX-2 and HO-1 protein levels were alleviated in microglial cells (Figure 3C). Therefore, our results and previous studies imply that paeonol-induced AMPK activation might be an important step in mediating the anti-inflammatory responses.

### 2.4. Paeonol-Induced Anti-Neuroinflammation is Mediated by GSK3α/β Activation

**Figure 4:**
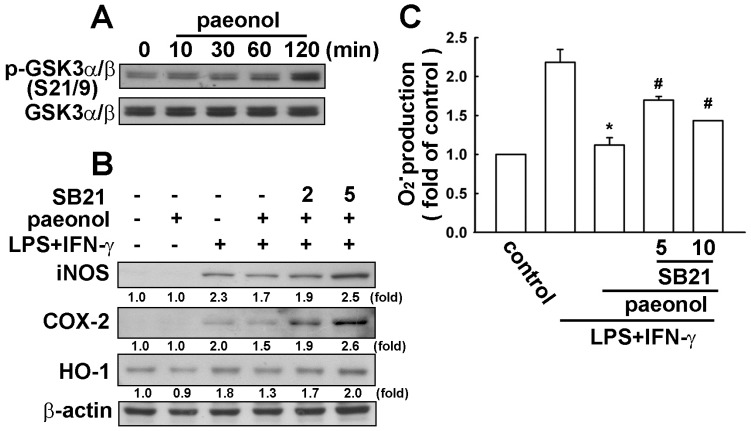
Involvement of GSK3α/β by paeonol in neuroinflammation. (**A**) BV-2 microglia cells were incubated with paeonol (10 μM) for indicated time periods (0–120 min). Whole-cell lysates were subjected to western blot analysis using the antibody against the phosphorylated GSK3α/β (Ser^21/9^); (**B**) Cells were pretreated with GSK3β inhibitor (SB216763; 2 or 5 μM) for 30 min and incubated with paeonol (10 μM) for another 30 min before LPS (10 ng/mL)/IFN-γ (10 ng/mL) treatment for 24 h. Whole cell lysis proteins were extracted and subjected to western blot for iNOS, COX-2 and HO-1 after incubation with LPS/IFN-γ. Similar results were obtained from at least four independent experiments; (**C**) Cells were pretreated with GSK3β inhibitor (SB216763) (5 or 10 μM) for 30 min and incubated with paeonol for another 30 min before LPS /IFN-γ treatment for 2 h; (**C**) O_2_^−^ generation was determined using the fluorescence probes DHE and then examined by flow cytometry. Results are expressed as the mean ± SEM of four independent experiments. * *p* < 0.05 compared with the control group. ^#^ *p* < 0.05 compared with the LPS/IFN-γ-treated group.

The role of GSK3β in mediating peripheral and central nervous system inflammation in a multitude of neurological disorders has been extensively studied. As shown in Figure 4A, stimulation of cells with paeonol increased GSK3α/β phosphorylation at the Ser^21/9^ site. We next determined whether the GSK3 inhibition is involved in paeonol-induced anti-neuroinflammation. Treatment of cells with GSK3 inhibitor SB216763 [32] inhibits GSK3α/β effectively reversed the inhibitory effects of paeonol on LPS/IFN-γ-induced iNOS, COX-2 and HO-1 protein levels (Figure 4B). Furthermore, treatment of GSK3 inhibitor SB216763 also dramatically reversed the inhibitory effects of paeonol on LPS/IFN-γ-induced ROS O_2_^−^ production (Figure 4C).

### 2.5. Effects of Paeonol on LPS-Induced Impairment of Motor Coordination and Microglial Activation

**Figure 5:**
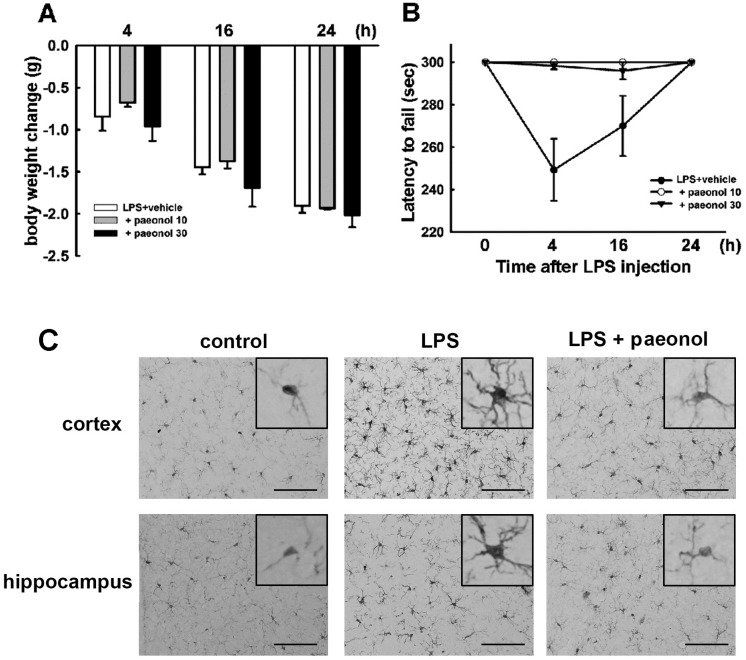
Effects of paeonol on LPS-induced motor coordination dysfunction and microglial activation. Mice were treated with paeonol and a single intraperitoneal injection of LPS. The body weight (A) and motor performance (B) were measured at 4, 16 or 24 h after LPS administration. Body weight change and latency to fall off rotard rod test for each animal group are shown as the mean ± SEM. All trials were performed three times for each animal; (**C**) The morphology of microglial activation was assessed by immunohistochemical analysis with the Iba-1-specific antibody in cortical and hippocampal regions. Scale = 100 μM.

It has been reported that systemic inflammation produced by intraperitoneal administration of LPS results in neuroinflammation-associated motor deficits. We investigated the effects of paeonol on LPS-induced body weight loss and motor coordination dysfunction. Increased body weight loss was observed in each animal group (Figure 5A). However, there was no difference of body weight loss between administration of paeonol and LPS-treated alone mice (Figure 5A). After LPS injection, decreased motor performance achieved a maximum at 4 h and sustained to 24 h. LPS-treated mice had shorter latency on the accelerating rotarod test, thus demonstrating motor impairments. However, treatment with paeonol significantly ameliorated these motor-impaired effects in LPS-injected mice (Figure 5B). The morphology of microglial activation was assessed by immunohistochemical analysis with the Iba-1-specific antibody. After LPS injection for twenty-four hours, microglia processes retracted, and cell bodies enlarged, showing more intensive immunoreactivities when compared with the control group. LPS stimulation induced pronounced hypertrophy of microglia, as microglial activation was observed homogeneously distributed among the cortical and hippocampal regions (Figure 5c). The microglial activation was protected by paeonol administration in mice in accordance with the motor performance experiment (Figure 5).

## 3. Discussion

Microglia is the vital element of the brain immune surveillance and the defense function in the brain [33,34,35]. Microglia are sensitive to external environment stimulation, and numerous reports showed that microglia immediately react to pathogenic stimuli by increasing the expression of innate inflammatory mediators [36]. Excessive inflammatory response has been found to be responsible for several neurodegenaerative diseases [36]. It is believed that microglia may play a role contributing to this process. However, it is important to maintain the balance of inflammatory responses to avoid overactive inflammatory responses. In order to maintain normal function of the CNS, activated microglia need to be tightly regulated.

Heme oxygenase (HO) is a rate-limiting enzyme that converses heme to CO, iron and biliverdin. Three HO isozymes have been identified: HO-1 is an inducible form that can be induced by oxidative stress, cellular injury, and disease [37]; HO-2 and HO-3 are constitutively expressed in various tissues [38]. It has been reported that HO-1 plays an important role in neuroprotection [39]. Our previous studies showed that induction of HO-1 expression exerts anti-neuroinflammation and neuroprotection in the CNS [40,41,42]. Our results showed that HO-1 plays a regulatory role in cytokine production, and we also reported the regulatory mechanisms of inflammation and anti-inflammation responses. Thus, HO-1 may act as an endogenous antioxidant protein to regulate inflammatory responses and oxidative states. Here, we demonstrate that paeonol regulated the HO-1 expression by acting as a counterbalance to the inflammatiory state.

Glycogen synthase kinase 3 α/β (GSK3α/β) is a serine/threonine kinase that regulates a diverse range of cellular functions including metabolism, cell survival and gene expression [43]. GSK3 activity is inhibited through phosphorylation of Ser 21 in GSK3 alpha and Ser 9 in GSK3 beta [44]. It has been demonstrated that inhibition of GSK3 protects cells against endotoxaemia [45], arthritis [46], and asthma [47] in mouse models. Moreover, GSK3α/β also plays a regulatory role in the CNS. It has been implicated in establishing axon formation [48], development and neuroplasticity [49]. Inhibition of GSK3α/β reduces plaques and tangles in mouse models of AD [50]. GSK3α/β inactivation has been viewed as a mechanism to promote neuronal survival [51]. Previous study showed that inactivation of GSK3β may be involved in its protective effect in microglia [52,53]. In our present study, paeonol inactivated GSK3α/β activity through phosphorylation of Ser 21 in GSK3 alpha and Ser 9 in GSK3 beta and subsequently contributed to inhibition of iNOS, COX-2 and ROS production. It has been reported that GSK3 negatively regulates AMPK function by interacted with the AMPK β regulatory subunit and directly phosphorylated the AMPK α subunit at threonine 479 [54]. Another study also demonstrated that AMPK activation increases GSK3β phosphorylation in the mouse liver [55]. AMPK activation seemed to increase the expression of peroxisome proliferators-activated response-coactivator-1α (PGC-1α), which may inhibit ROS production in mitochondria [56]. AMPK and GSK3α/β have been indicated to regulate inflammation and eliminate ROS production. These studies indicate that AMPK plays an important role in anti-neuroinflammation. Here, we further examined the involvement of paeonol in the AMPK and GSK3α/β signaling pathways.

Molecular inflammatory responses and sickness-like behaviors have been well characterized in neuroinflammtory-mediated mice. Exogenous inflammatory mediators induce sickness behaviors such as anorexia, decreased locomotion, and social activity, whereas inhibition of inflammatory signaling attenuates sickness behaviors in response to LPS treatment [57,58]. It has been reported that paeonol has an anxiolytic-like effect [59] and increases learning performance [60]. In the present study, we further examined the effect of paeonol on inflammation-mediated motor coordination. We performed an intraperitoneal LPS injection in mice to mimic systemic infections that can produce exaggerated behavioral impairment and microglial activation. After LPS injection, each animal group had increased body weight loss, however, there was no differences between administration of paeonol and LPS-treated alone mice. Nevertheless, paeonol significantly rescued the latency to fall of rotarod and microglial activation caused by LPS induction. For these reasons, we suggested that paeonol would exert an anti-inflammatory benefit by inhibiting neuroiflammation, and this would attenuate LPS-induced motor dysfunction in the paeonol treatment group. Therefore, paeonol may be beneficial for improving neuroinflammation-induced motor impairment.

Taken together, our findings suggest that paeonol activates AMPK, and GSK3α/β subsequently inhibits inflammatory signaling thus contributing to anti-neuroinflammation. Our results also support previous reports demonstrating that AMPK activation attenuates inflammatory responses and this offers new insight for an alternative approach to the development of novel drugs based on inhibition of inflammatory signaling pathways to treat anti-inflammatory-related disorders.

## 4. Experimental Section

### 4.1. Reagents and Antibodies

Paeonol and primary antibodies against β-actin, ERK2, p38, JNK, GSK3α/β, phosphor-ERK1/2, phosphor-p38 and phosphor-JNK were purchased from Santa Cruz Biotechnology (Santa Cruz, CA, USA). Adenine 9-beta-d-arabinofuranoside (Ara-A) and SB216763 were purchased from Calbiochem (San Diego, CA, USA). The HO-1 antibody was purchased from StressGen Biotechnologies (San Diego, CA, USA). Primary antibodies against AMPK (phosphorylated at Thr^172^), GSK3α/β (phosphorylated at Ser^21^and Ser^9^) and LKB1 (phosphorylated at Ser^428^) were purchased from Cell Signaling and Neuroscience (Danvers, MA, USA). The primary antibody against iNOS was purchased from BD Transduction Lab (Lexington, KY, USA). The primary antibody against COX-2 was purchased from Cayman Chemicals (Ann Arbor, MI, USA).

### 4.2. Cell Culture

The murine microglial cell line BV-2 was originally generated by infecting primary microglial cell cultures with a v-raf/v-myc oncogene carrying a retrovirus (J2). Since BV-2 cells retain most of the morphological, phenotypical, and functional properties described for freshly isolated microglial cells, they can be considered as immortalized active microglial cells. Cells were cultured in DMEM (Gibco, Grand Island, NY, USA) with 10% FBS at 37°C, and passaged by trypsinization.

### 4.3. Animals

All mice were manipulated in accordance with the Animal Care and Use Guidelines of the China Medical University (Taichung, Taiwan). Eight-week-old male imprinting control region (ICR) mice were purchased from the National Laboratory Animal Center (Taipei, Taiwan). The animals were housed in a temperature- and humidity-controlled environment and given access to food and water *ad libitum*. Mice were acclimated to their environment for seven days before the experiments.

### 4.4. Western Blot Analysis

Cells were lysed briefly in homogenizing buffer [61] for 30 min on ice. Equal amounts of the samples were loaded in each lane. The membranes were blocked with non-fat milk in phosphate-buffered saline (PBS) and then probed with primary antibodies. After undergoing three PBS washes, the membranes were incubated with secondary antibodies. The blots were visualized by enhanced chemiluminescence using Kodak X-OMAT LS film (Eastman Kodak, Rochester, NY, USA).

### 4.5. Migration Assay

*In vitro* migration and invasion assays were performed using Costar Transwell inserts (Costar, NY, USA; pore size, 8 μm) in 24-well plates as described previously [62,63]. Approximately 1 × 10^4^ cells in 200 μL of serum-free medium were placed in the upper chamber, and 300 μL of the same medium containing ATP was placed in the lower chamber. Before performing the migration assay, cells were pre-treated for 30 min with paeonol followed by treatment with ATP for 24 h. The plates were incubated for 24 h at 37 °C in 5% CO_2_, and then cells were stained with 0.05% crystal violet and 2% methanol. Non-migratory cells on the upper surface of the filters were removed by wiping with a cotton swab. The cell number in three fields per well was counted under a microscope at 100× magnification. Images of migratory cells were observed and acquired with a digital camera and light microscope.

### 4.6. Reactive Oxygen Species (ROS) Assay

The production of intracellular O_2_^−^ and hydrogen peroxide (H_2_O_2_) were assessed spectrofluorimetrically by oxidation of specific probes dihydroethidium (DHE) and 2',7'-dichlorodihydrofluorescein diacetate (H_2_DCFDA) according to our previous studies [64,65]. Cells were plated on six-well plates and pre-treated for 30 min with paeonol followed by treatment with LPS + IFN-γ for 24 h. The cells were incubated with DHE (10 μM) or H_2_DCFDA (10 μM) for 30 min at 37 °C. The fluorescence intensity was measured with an excitation filter of 488 and 525 nm emission wavelengths using flow cytometry (BD Biosciences, San Jose, CA, USA).

### 4.7. Sickness-Like Behaviors

Mice were treated with paeonol (10 or 30 mg/kg) or vehicle intraperitoneally once daily for three consecutive days before a single intraperitoneal injection with LPS (5 mg/kg; *E. coli*, serotype 0127:B8). Body weight and motor performance were recorded 4, 16 and 24 h after LPS injection. Motor balance and coordination function were analyzed using an UgoBasile 7650 accelerating rotarod (Linton Instruments, Diss, UK). The treadmill was accelerated from 20 to 60 rpm over a period of 5 min, and the time spent on the drum was recorded for each mouse. Once the mice were placed on the rotating drums, the counter was started, and the rod was set to accelerate after 30 s. Any mouse remaining on the apparatus after 5 min was removed, and its time was scored as 5 min. Latency to fall was calculated in seconds and used for data analysis.

### 4.8. Immunohistochemical Analysis

Tissue preparation and immunohistochemistry were performed according to our previous reports [66,67]. After performing rotarod tests, mice were deeply anesthetized, transcardially perfused with 10% formaldehyde, and brains were removed and post-fixed overnight. Brain samples were stored in a 30% sucrose solution at 4 °C. ABrain slices were first treated with 0.3% hydrogen peroxide for 15 min to remove the endogenous peroxidase. Brain sections were then incubated with Triton X-100. After blocking with bovine serum albumin, the slices were incubated with primary antibody against Iba-1 (Wako Pure Chemicals, Osaka, Japan) for microglia staining. Binding was detected using a biotinylated secondary antibody and an avidin-biotin complex kit (Vector Laboratories, Burlingame, CA, USA), followed by using diaminobenzene (Sigma-Aldrich, St. Louis, MO, USA) as the chromogen and acquired with a digital camera and light microscope [68].

### 4.9. Measurement of Cell Viability

Cell viability was assessed by the 3-(4,5-dimethylthiazol-2-yl)-2,5-diphenyltetrazolium bromide (MTT) assay as described previously [69]. Briefly, cells were treated with various concentrations of paenol for 24 h. The culture medium was removed and MTT reagent was added, and cells were dissolved in dimethyl sulfoxide. The absorbency values were measured in a microplate reader.

### 4.10. Statistical Analyses

Results were analyzed using GraphPad Prism software version 5 (Graph Pad software Inc., San Diego, CA, USA) and is expressed as means ± S.E.M. Significant differences between two groups were assessed by the Student’s *t*-test, and multiple comparisons were compared by one-way ANOVA analysis of variance followed by Tukey *post hoc* test. The difference was determined to be significant if the *p* value was <0.05.

## 5. Conclusions

The present study demonstrates that paeonol significantly induces activation of AMPKα and GSK3α/β signaling pathways to inhibit inflammatory and oxidative mediators. Furthermore, paeonol inhibits LPS/INF-γ-induced p38 and STAT3 signaling pathways in microglial cells. *In vivo* results also show that paeonol significantly improves LPS-induced motor coordination dysfunction. Our data demonstrates a key signaling pathway elicited through paeonol in neuroinflammatory responses.
